# A case of internal hernia in the pararectal fossa

**DOI:** 10.1186/s40792-023-01746-0

**Published:** 2023-10-16

**Authors:** Kozue Matsuishi, Seiya Saito, Mayuko Ohuchi, Yuki Kiyozumi, Jiro Nasu, Norihisa Hanada, Hideo Baba

**Affiliations:** 1 Department of Surgery, Kumamoto Chuo Hospital, 1-5-1 Tainoshima, Minami-Ku, Kumamoto, 862-0965 Japan; 2 Department of Surgery, Izumi General Medical Center, 520 Myozincho, Izumi City, Kagoshima 899-0131 Japan; 3 Department of Gastroenterological Surgery, Graduate School of Medical Sciences, Kumamoto University, 1-1-1 Honjo, Chuo-Ku, Kumamoto, 860-8556 Japan

**Keywords:** Pararectal fossa hernia, Internal hernia, Intestinal obstruction, Perirectal fossa hernia

## Abstract

**Background:**

Internal hernias are relatively rare and difficult to diagnose. Diagnostic delays lead to the progression of strangulation. In particular, pararectal fossa hernias are extremely rare. We encountered a case in which internal hernia occurred in the pararectal fossa.

**Case presentation:**

An 87-year-old woman was referred to our hospital because of persistent lower abdominal pain and vomiting. Contrast-enhanced computed tomography revealed findings of intestinal ischemia, such as closed loop formation with reduced contrast effect on the left side of the rectum in the pelvis. Strangulation small bowel obstruction was diagnosed, and emergency laparotomy was performed. The small intestine was found to invade the peritoneal reflection on the left side of the rectum. The patient was finally diagnosed with pararectal fossa hernia. The incarcerated small intestine was released with no bowel resection. The 4-cm hernia phylum was observed and closed by simple suture. The patient had a good postoperative course without recurrence.

**Conclusions:**

We encountered a very rare case of internal hernia in the left pararectal fossa. Preoperative diagnosis of this disease is difficult, but it should nevertheless be considered in cases in which the cause of the intestinal obstruction is unknown.

## Background

Internal hernia is a rare condition that causes intestinal obstruction. It is defined as the herniation of an organ into an unusually large fossa, fovea, or foramen in the abdominal cavity [1]. Internal hernias are reported to comprise 0.2–5.8% of all cases of small bowel obstruction, which itself is a rare disease [2]. The diagnosis of internal hernia is difficult, and diagnostic delays lead to the progression of strangulation [3]. Particularly, among cases of internal hernia, cases of pararectal fossa hernias are extremely rare. We encountered a rare case in which internal hernia occurred in the pararectal fossa. Herein, we report the case with a review of the literature.

## Case presentation

**Fig. 1:**
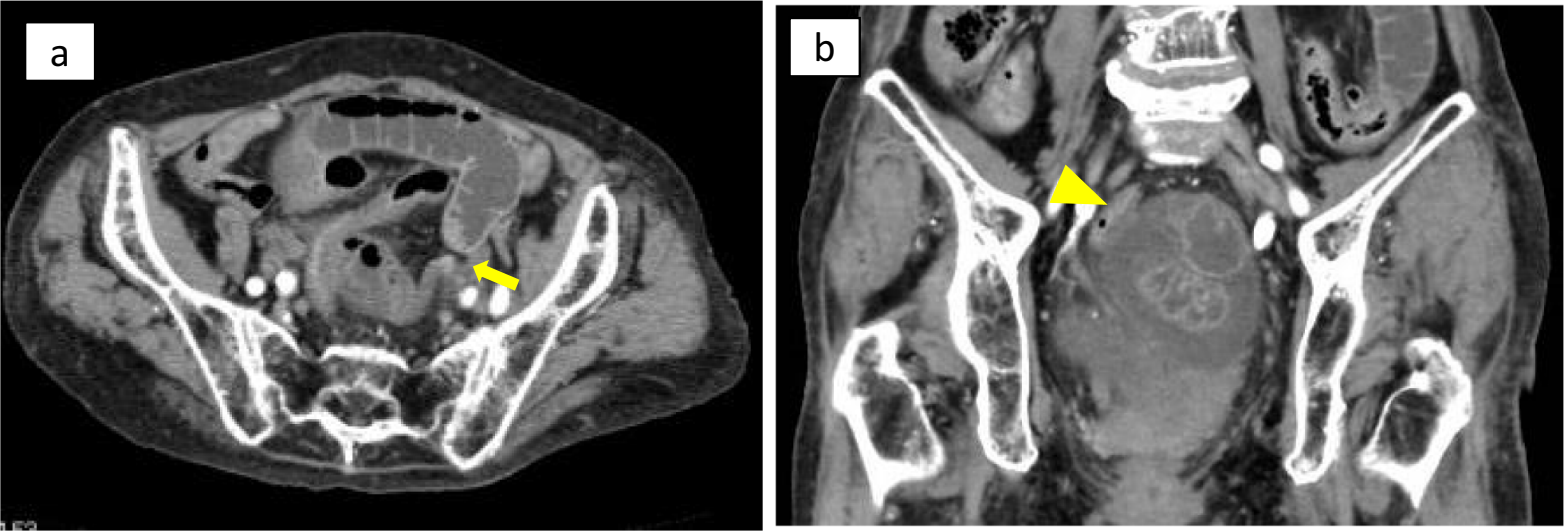
Computed tomography before treatment. **a** Abdominal computed tomography shows extensive small intestinal dilation and beak-like changes in the small intestine (arrow) on the left side of the pelvis. **b** A closed loop with reduced contrast effect is formed on the left side of the rectum (arrow head), and localized ascites is observed

**Fig. 2:**
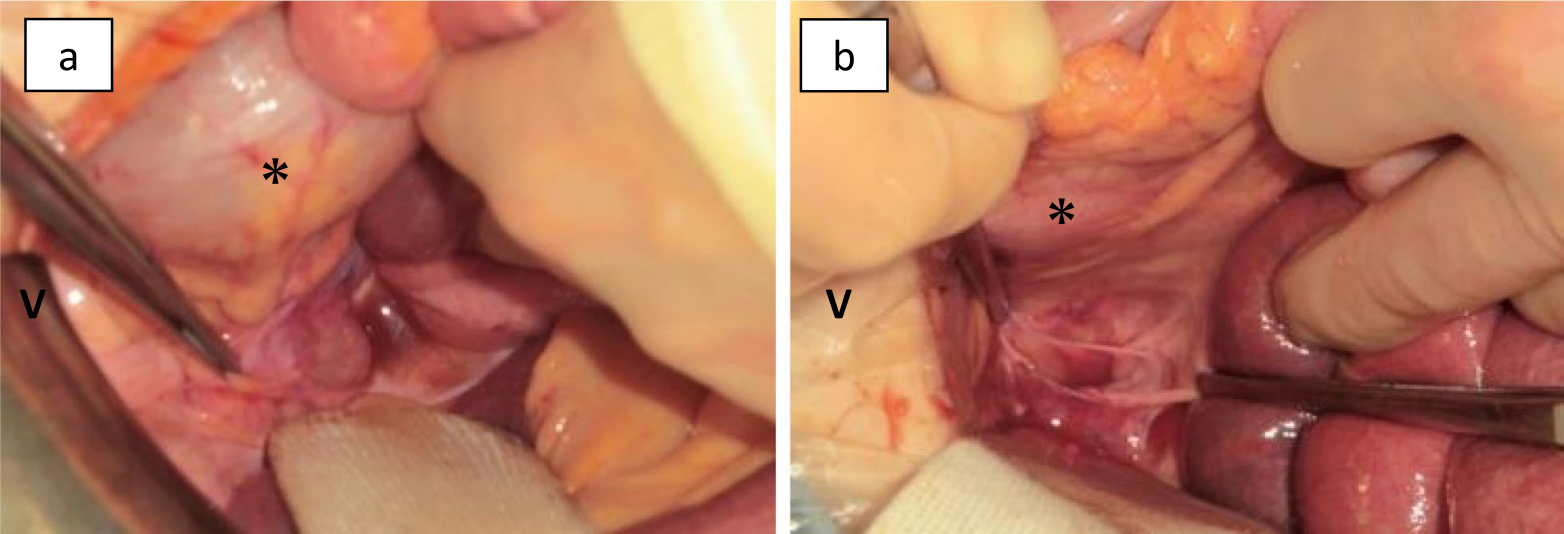
Intraoperative images. **a** The small intestine was stuck in the pararectal fossa on the left side of the rectum (asterisk). V = ventral side. **b** A 4-cm hernia orifice was found in the peritoneal reflection on the left side of the rectum (asterisk)

An 87-year-old woman presented with the chief complaint of lower abdominal pain and nausea. She was referred to our hospital with persistent lower abdominal pain and vomiting. On physical examination, mild tenderness was present in the lower abdomen. Blood test revealed elevated white blood cell count (12,200 /μl) and C-reactive protein levels (2.2 g/dl), with no other pertinent findings. The patient had diabetes and high blood pressure and had previously undergone total hysterectomy for uterine fibroids. Contrast-enhanced computed tomography showed beak-like changes in the small intestine, closed loop formation with reduced contrast effect, and localized ascites on the left side of the rectum in the pelvis (Fig. 1a, b). Strangulation small bowel obstruction was diagnosed, and emergency laparotomy was performed. The small intestine was found to invade the peritoneal reflection on the left side of the rectum (Fig. 2a, b). The final diagnosis was pararectal fossa hernia. The incarcerated small intestine was released, and no bowel resection of the same site was required because the intestinal blood flow disorder was reversible. The 4-cm hernia phylum was observed and closed by simple suture (Fig. 2b). The patient had a good postoperative course and no recurrence.

## Discussion

Internal hernia is defined as the herniation of an organ into an unusually large fossa, fovea, or foramen in the abdominal cavity [1]. Internal hernias occur in 0.2–5.8% of all cases of small bowel obstruction [2, 4]. Particularly, paraduodenal fossa hernias are reported to be the most common (53%), followed by paracecal fossa hernias (13%), and Winslow foramen hernias and transverse mesenteric fossa hernias (8%) [3]. On the other hand, internal hernias in the pelvis, including the broad ligament of the uterus, Douglas fossa, and pararectal fossa, occur in < 6% of cases [3]. Furthermore, there are several cases of the broad ligament of the uterus reported as internal hernias in the pelvis [5, 6]. Thus, it can be concluded that the occurrence of internal hernia in the pararectal fossa is extremely rare.

A search on the PubMed and MEDLINE databases for literature published regarding “pararectal fossa hernia” or “perirectal fossa hernia” yielded a total of three studies [3, 7, 8] (Table 1). All previous cases were in middle-aged women, but our case was in a very elderly woman. In two cases, internal hernia occurred on the right side of the rectum, whereas in the other two (including our case), it occurred on the left side of the rectum. The latter two cases had a history of hysterectomy. The cause may be congenital or acquired, but congenital factors were suspected in the cases in which internal hernia occurred on the right side of the rectum considering the early age of onset and the absence of a history of surgery. On the other hand, the cases in which internal hernia occurred on the left side of the rectum were presumed to be associated with a history of gynecological surgery.

**Table 1:** Previously reported cases of pararectal fossa hernia

No.	Author	Reported year	Age (y)	Sex	History of surgery	Position	Symptom	Hernia content	Surgical approach	Procedure
1	Takeyama [3]	2005	28	Female	–	Right	Ileus	Bowel	Laparotomy	Suture
2	Yamashiro [7]	2007	48	Female	–	Right	Ileus	Bowel	Laparoscopy	Not detected
3	Walid [8]	2010	48	Female	Total hysterectomy	Left	Incidental	No content	Laparoscopy	Mesh
4	Our Case	2021	87	Female	Total hysterectomy	Left	Ileus	Bowel	Laparotomy	Suture

A differential disease of pararectal fossa hernia includes pararectal hernia, a type of perineal hernia. Perineal hernia is defined as a pathological condition in which intra-abdominal organs protrude beyond the pelvic floor into the subcutaneous area of the perineum. A defect is found in the pelvic floor, which is composed of pelvic floor muscles, and it is considered to be a condition in which a hernia orifice is formed [9]. Internal hernias in the pelvis, including this case, are caused by defects up to the peritoneum, and should be distinguished from perineal hernias.

In this case, we performed laparotomy to release the strangulation and closed the hernia phylum with suture. In recent years, there have been cases in which laparoscopic surgery was performed for strangulated small bowel obstruction [10], and both laparoscopic surgery and laparotomy are considered as treatment options. Thus, it is important to determine which operative procedure is better based on the patient’s condition, including the status of small bowel obstruction, such as dilation of the intestinal tract.

Closure of the hernia phylum involves the use of ligation sutures and a repair method using a mesh [3, 8]. If the intestinal tract is necrotic, it is difficult to use the mesh owing to the possibility of infection. Thus, it is necessary to choose the appropriate closure method for hernia phylum based on the patient’s condition.

## Conclusion

We present a case of pararectal fossa hernia, which is a very rare disease among the different types of internal hernias. Preoperative diagnosis is difficult, but a differential diagnosis of pelvic internal hernia should nevertheless be considered. In such cases, early surgery is desirable.

## Data Availability

All data generated or analyzed during this study are included in this published article.
